# Management of HAM/TSP: Systematic Review and Consensus-based Recommendations 2019

**DOI:** 10.1212/CPJ.0000000000000832

**Published:** 2021-02

**Authors:** Abelardo Araujo, Charles R.M. Bangham, Jorge Casseb, Eduardo Gotuzzo, Steve Jacobson, Fabiola Martin, Augusto Penalva de Oliveira, Marzia Puccioni-Sohler, Graham P. Taylor, Yoshihisa Yamano

**Affiliations:** Laboratory for Clinical Research in Neuroinfections (AA), Evandro Chagas National Institute of Infectious Diseases, FIOCRUZ, Rio de Janeiro, Brazil; Section of Immunology of Infection (CRMB), Department of Infectious Disease, Imperial College London, United Kingdom; Faculdade de Medicina da Universidade de São Paulo/Institute of Tropical Medicine of Sao Paulo (JC), Brazil; Instituto de Medicina Tropical “Alexander von Humboldt” (EG), Universidad Peruana Cayetano Heredia, Lima, Peru; Viral Immunology Section (SJ), National Institutes of Health, Bethesda, MD; Stonewall Medical Centre (FM), Windsor, Australia; Instituto de Infectologia Hospital Emilio Ribas (APO), Sao Paulo University, Sao Paulo, Brazil; Federal University of the State of Rio de Janeiro (UNIRIO)/Federal University of Rio de Janeiro (UFRJ) (MP-S), Brazil; Section of Virology (GPT), Department of Infectious Disease, Imperial College London, United Kingdom; and Department of Rare Diseases Research (YY), Institute of Medical Science, St Marianna University School of Medicine, Kanagawa, Japan.

## Abstract

**Purpose of Review:**

To provide an evidence-based approach to the use of therapies that are prescribed to improve the natural history of HTLV-1–associated myelopathy/tropical spastic paraparesis (HAM/TSP)—a rare disease.

**Recent Findings:**

All 41 articles on the clinical outcome of disease-modifying therapy for HAM/TSP were included in a systematic review by members of the International Retrovirology Association; we report here the consensus assessment and recommendations. The quality of available evidence is low, based for the most part on observational studies, with only 1 double-masked placebo-controlled randomized trial.

**Summary:**

There is evidence to support the use of both high-dose pulsed methyl prednisolone for induction and low-dose (5 mg) oral prednisolone as maintenance therapy for progressive disease. There is no evidence to support the use of antiretroviral therapy. There is insufficient evidence to support the use of interferon-α as a first-line therapy.

At a conservative estimate, 5–10 million persons are infected with HTLV-1 globally.^1^ HTLV-1–associated myelopathy/tropical spastic paraparesis (HAM/TSP) occurs in ∼3% of HTLV-1 carriers. The risk varies between different endemic regions: the lowest reported lifetime risk is 0.25% from Japan, whereas data from Brazil indicate a risk much higher than 3%, with an incidence of 1.47% over a median of 3 years in 1 cohort.^2,3^


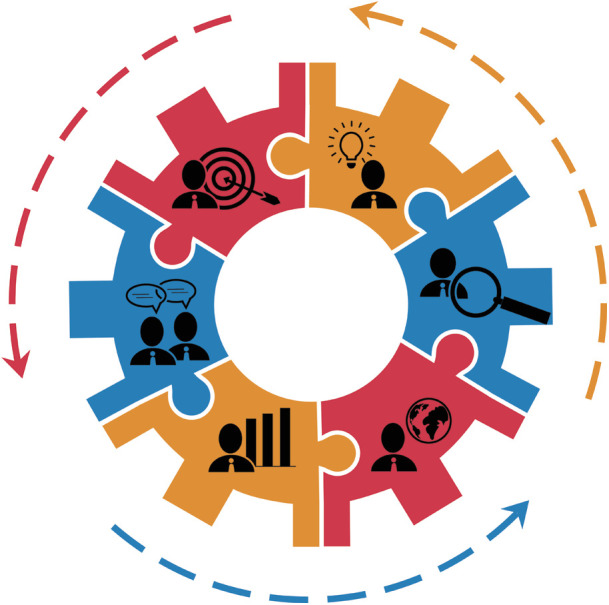


Although the range of symptoms can be extensive, there are 5 cardinal symptoms: lower limb stiffness and/or weakness; lumbar pain with or without radiation; bladder dysfunction (spastic or flaccid); bowel dysfunction, usually presenting as constipation; and sexual dysfunction. The neurologic findings are reported in detail elsewhere.^4^ Natural history studies indicate a chronic progressive deterioration, resulting in 50% of patients with HAM/TSP becoming wheelchair dependent within 20 years of first symptoms. Rates of progression vary widely, with a subset remaining stable over many years while a small minority become rapidly bedbound.^5^

Although symptomatic management and physical therapies can improve function and quality of life in HAM/TSP, they do not alter the natural history of the condition. In this guideline, published on behalf of the International Retrovirology Association (IRVA) for the management of patients with HAM/TSP, the potential of disease-modifying therapies (DMTs) is reviewed. The term DMT will be used in this review to refer to any therapy, which targets the disease process, be it anti-inflammatory or anti-viral, rather than symptomatic treatment. A broad range of compounds have been examined over the past 3 decades, but mostly in observational studies, and there are no published guidelines on their use.

## Methods

An international panel of physicians and clinical scientists from neurology and infectious diseases, experienced in the management of patients with HAM/TSP, was convened from the membership of the International Retrovirology Association, the association for research, education, and training on HTLVs and associated diseases.

Two approaches were taken. First, a literature review was conducted by searching PubMed in January 2017 using the terms HTLV-1-associated myelopathy, tropical spastic paraparesis, therapy and treatment. Second, the biennial conference proceedings of IRVA were systematically reviewed. The disposition of articles is presented in the figure. Studies were included if they reported an observational cohort, an open-treatment study or a randomized controlled study (masked or unmasked), with 1 or more clinical outcome measures. Case reports and studies of surrogate markers were excluded. The outcomes of interest were changes in disability, mobility, pain, bladder, or bowel function in relation to therapy directed at the underlying disease, rather than symptomatic management. The preferred measure of effect was the time taken to walk a fixed distance (typically 10 m). None of the studies presented confidence intervals, and only 2 studies were prospective randomized studies, both of small size. Important clinical effects were any measured change in time taken to walk 10 m, change in validated disability scale, or change in pain as measured on a visual analogue scale. The findings of the systematic review were presented for consultation at an open workshop held during the 18th International Conference on Human Retrovirology: HTLV and related viruses (March 2017) Kamakura, Japan. Thereafter, panel members met on 2 occasions to formulate recommendations. The draft recommendations were circulated to and commented on by all panel members with the addition of any eligible studies published since the original literature search. The resulting recommendations, made in accordance with the GRADE Guidelines,^6^ represent the consensus reached by discussion among the panel members. The draft recommendations were presented at the IRVA Tokyo Conference and International Symposium on July 13, 2018, and then published on the International Retrovirology Association web site for a period of public consultation. The final recommendations were presented at the 19th International Conference on Human Retrovirology: HTLV and related viruses (Lima, Peru, April 24–26, 2019).

**Figure:**
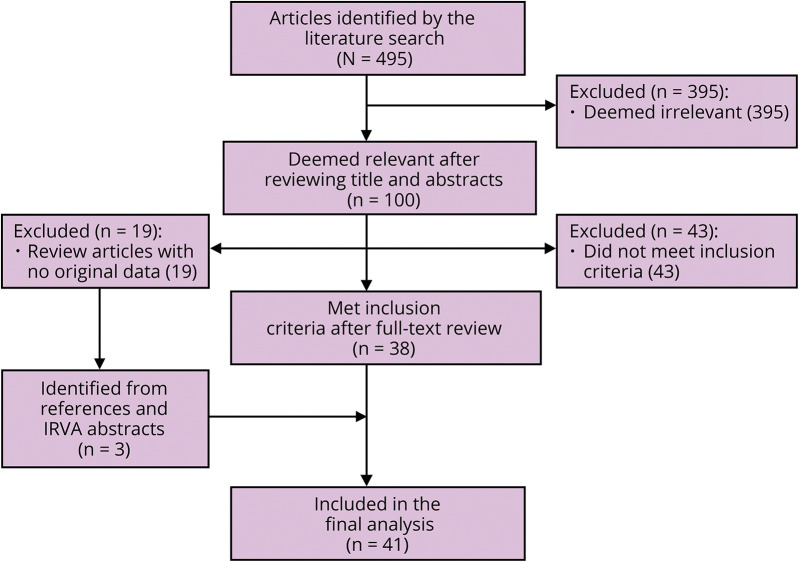
Flow Diagram Documenting the Disposition of Articles During the Systematic Review

### Data Availability

All data used to inform these recommendations are published and are summarized and cited in appendix e-1, links.lww.com/CPJ/A170.

## Recommendations and Context

1. It is recommended that clinical studies of therapy for HAM/TSP should predefine patients into the following categories: rapid progression, slow progression, and very slowly or nonprogressing, and report outcomes separately for each category.

HAM/TSP has a broad spectrum of severity and consequently the potential benefit of therapies that aim to modify the progression of the disease varies considerably. The natural history of HAM/TSP ranges from a disease that renders the patient bedbound within months to minor disturbances of gait or abnormalities of bladder function that remain stable over many years (table).

**Table:**
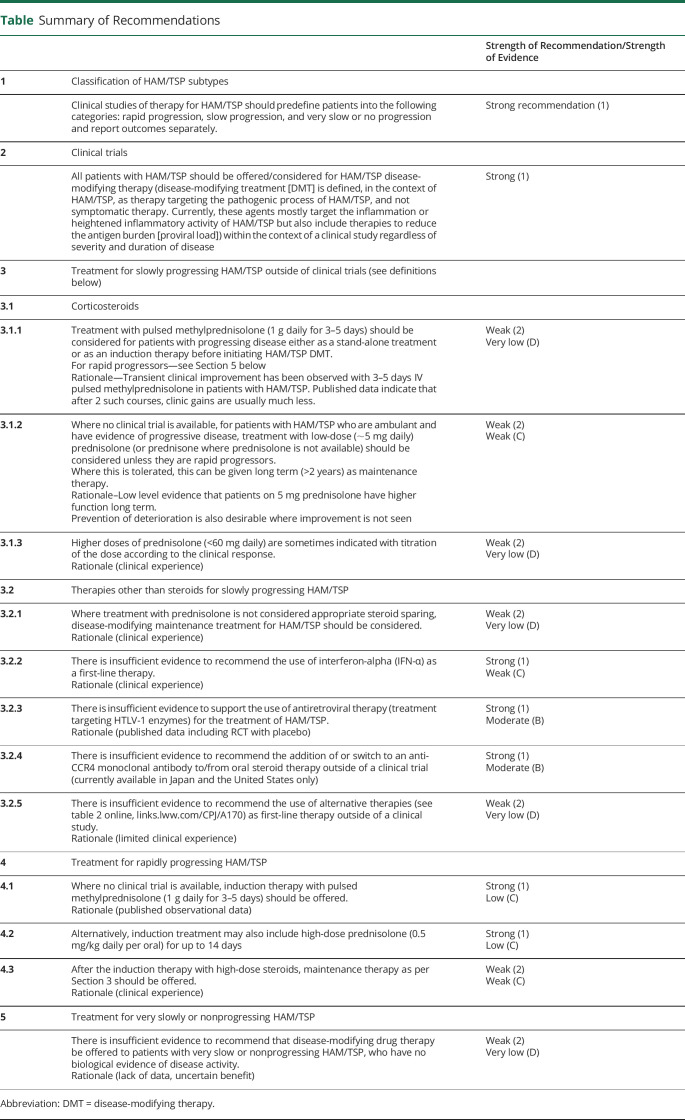
Summary of Recommendations

There is increasing evidence that responsiveness to therapy (reduced symptoms and increased mobility) correlates with the duration and stage of disease. Because current therapies that aim to alter the course of HAM/TSP all have notable risks, it is important to select those patients who are most likely to benefit. Patients with rapidly progressing disease should be treated immediately. In addition, patients with rapid disease may require more intense treatment to modify disease progression. Clinical experience suggests that more aggressive therapy that would not be considered for patients with slowly progressing disease can restore mobility in rapidly progressing patients. However, this suggestion needs to be verified through clinical studies.

The optimal management of the milder forms of HAM/TSP will be discussed in detail. However, the relative merits of treatment need to be determined for each clinical subgroup of patients to ensure that the risks and benefits are appropriately assessed.

### Rapid Progression

In the ongoing randomized controlled study HAMLET-p, comparing placebo with prednisolone, patients are defined as rapid progressors if they present with or display 1 or more of the 3 deterioration criteria in the clinical history or all 4 deterioration criteria in the clinical examination at screening visit or during the 3-month assessment period:

#### Criteria of Rapid Deterioration

1. Clinical history
  A) In the preceding 3 months:
    • Loss of ability to run.
    • Loss of ability to climb stairs unaided (now needing to use at least 1 banister to climb up or downstairs).
    • First symptoms of HAM/TSP during the preceding 3 months and already needs a walking aid (unless this need is unrelated to HAM/TSP).
  B) In the preceding 2 years:
    • Progression from walking unaided to wheelchair dependent or bedbound within 2 years of onset of symptoms
2. Documented clinical examination during the following 3 months
  • Additional walking aid needed
  • Increase in 10-m timed walk (seconds) by ≥30%
  • Decrease in 6-minute timed walk (minutes) ≥30%
  • Increase in timed up and go (seconds) ≥30%

### Slow Progression

Patients are defined as slow progressors if they meet none of the rapid progression criteria listed above, but 1 or more of the 3 clinical examination criteria for slow progression, at any point during the assessment period:

#### Criteria of Slow Progression

Documented by clinical examination over a 3-month period,

• Increase in 10-m timed walk (seconds) by ≥10%
• Decrease in 6-minute timed walk (minutes) ≥10%
• Increase in timed up and go (seconds) ≥10%

Outside a clinical trial, clinicians may choose to estimate the rate of progression from the history, but this can make interpretation of the clinical response more difficult.

#### Very Slow Progression (or No Progression)

Patients who do not meet any of the clinical history or clinical examination criteria of slow or rapid progression listed above.

A few patients show very slow progression of motor disability. For example, a patient may lose the ability to run at 10 years or more after the onset of motor symptoms, but still can climb up or downstairs without any support.^5^

Conclusion/expert opinion: Although these definitions require international validation in multiple settings before becoming standardized in clinical trials, they are noninvasive and easily assessed. The definitions are presented here to identify patients who would benefit from the treatments outlined below, while recognizing that local variations are used. It is helpful to use more than 1 clinical measure: for example, in patients with HAM/TSP, the 10-m timed walk has been shown to detect change but underestimate fatigue, which is identified with the 6-minute timed walk.^7^

2. All patients with HAM/TSP should be considered for and offered disease-modifying therapy within the context of a clinical study, regardless of severity and duration of disease.

Currently, clinical trials for patients with HAM/TSP are uncommon. However, there is an urgent need for higher quality evidence to support any recommendations because, as is shown below, the evidence base for guiding treatment for patients with HAM/TSP is extremely limited. Because the potential effect on any patient of the current (and future) therapies is uncertain, the safety and efficacy of any therapy need to be tested across the spectrum of disease severity.

3. Therapy for patients with slowly progressing HAM/TSP

3.1. Use of corticosteroids

Before starting any immunosuppressive therapy, patients with HTLV-1 infection should be screened for HIV, hepatitis B and C, syphilis, *Strongyloides stercoralis* (if they are or were resident in an endemic region), and tuberculosis and treated as appropriate. Other clinical contraindications to the use of corticosteroids in the short or long term must also be considered, and adult T-cell leukemia/lymphoma must be excluded.

3.1.1. Treatment with pulsed methylprednisolone (1 g daily for 3–5 days) should be considered for patients with progressing disease either as a stand-alone treatment or as an induction therapy before starting HAM/TSP disease-modifying therapy.

Conclusion/expert opinion: Pulsed methylprednisolone is well tolerated, but is associated with only transient clinical improvement, mainly in movement or pain. The effects are seen within days and persist for several months in a proportion of patients. One study indicated that the benefits may be maintained by repeated courses, but more data are required on treatment with more than 2 courses. Treatment in earlier disease tends to achieve better results. The expert panel considered that pulsed methylprednisolone can be an effective approach to initiating disease-modifying therapy for slowly progressing HAM/TSP.

3.1.2. For patients with HAM/TSP who are ambulant and have evidence of progressive disease, treatment with low-dose (∼5 mg daily) prednisolone (or prednisone where prednisolone is not available) can be offered unless they are rapid progressors. Where this is tolerated, this can be given long term (up to 4 years) as maintenance therapy.

3.1.3. Higher doses of prednisolone (<60 mg daily) are sometimes indicated with titration of the dose according to the clinical response.

Conclusion/expert opinion: The most recent evidence emphasizes the ongoing deterioration seen in untreated slowly progressing HAM/TSP and suggests that low-dose (∼5 mg) prednisolone daily for at least 4 years can give clinical benefit. There remains considerable uncertainty over the optimal duration of treatment, and long-term studies of adverse events are required. In these nonrandomized studies, there might have been a bias toward treatment, especially if patients were deteriorating at baseline, which might mask some of the benefits, and higher-risk patients (those with osteopenia, diabetes mellitus, hypertension, etc.) might have been less likely to receive treatment. The consensus was that there is sufficient evidence, albeit of low quality, to consider 5 mg prednisolone daily for up to 4 years as first-line therapy for patients with slowly progressing HAM/TSP and without contraindications. As this may not apply to all patients due to individual circumstances, the recommendation is only weak, allowing individualization of recommendations. Because benefit is seen at 5 mg daily, patients started on higher doses should aim to reduce to this dose as far as possible.

The benefits of prednisolone for very slow progressors or nonprogressors are unknown, and a watchful waiting approach is recommended. First-line therapy for patients who are progressing rapidly is addressed in Section 4.

3.2. Therapies other than corticosteroids

3.2.1. Where treatment with prednisolone is not considered appropriate, the offer of steroid-sparing, anti-inflammatory, disease-modifying maintenance treatment for HAM/TSP should be considered.

Conclusion/expert opinion: The effect of various steroid-sparing, anti-inflammatory therapies has been reported in patients with HAM/TSP in a mixture of retrospective and prospective studies. The studies generally report a favorable clinical response, but the numbers are small, and, with the exception of ciclosporin, which was given for 48 weeks, the duration of treatment was short (1–3 months). More studies are required to determine the role of steroid-sparing therapy in the treatment of patients with HAM/TSP, particularly in patients with contraindications to prednisolone or where a response is not maintained at 5–10 mg daily. In such patients, steroid-sparing immunosuppressive therapies should be considered case by case.

3.2.2. There is insufficient evidence to recommend the offer of interferon-alpha (IFN-α) as a first-line therapy.

Conclusion/expert opinion: Clinical improvement is observed in some patients treated with 3 MIU interferon-α for up to 4 weeks. However, side effects are common. There are insufficient data on treatment beyond 4 weeks, and data from 2 studies suggest that even where improvement at 4 is maintained at 6 months, the benefit is gradually lost once treatment is discontinued. Although interferon-α has been licensed for the treatment of HAM/TSP in Japan since January 2000 (whereas prednisolone is not licensed there), in a recent survey, only 2–3% of patients with HAM/TSP in Japan are currently treated with interferon.^8^ The panel concluded that the quality of the evidence on efficacy was low, that intolerance was high, and that although 1 RCT showed moderately good evidence of short-term improvement, the current data do not support the use of interferon-α in patients with HAM/TSP either as first-line therapy or in long-term treatment.

Although not consistently reported, in a number of studies, response rates appear to be better with milder disease and shorter duration of symptoms. Future studies should be powered to include disease severity categories to ensure that potential benefits are not missed through treating patients too late and that patients with late-stage disease are not unnecessarily exposed to potential toxic therapies.

3.2.3. There is insufficient evidence to support the offer of antiretroviral therapy (treatment targeting HTLV-1 enzymes) for the treatment of HAM/TSP.

Conclusion/expert opinion: HAM/TSP is associated with a high HTLV-1 viral burden. Given the similarities in life cycle of HTLV-1 to HIV, the potential of antiretroviral therapy to reduce HTLV-1 proviral load, with the anticipated prospect of reduced inflammation, has been tested. These studies have demonstrated that HTLV-1 proviral load in established infection is not reduced by HTLV-1 enzyme inhibitors. The investigation of HTLV-1 replication in vivo clearly identifies the importance of virus-driven proliferation of infected cells in both primary and chronic infection. Although some degree of infectious spread is likely to continue in chronic infection, its relative contribution to proviral load is small, accounting of the lack of effect of antiretroviral therapy. The distinct potential role of antiretroviral therapy to prevent infection is not addressed here. The combination of antiretroviral therapy with a histone deacetylase inhibitor, sodium valproate, which markedly reduced STLV-1 proviral load in baboons, has not been tested in humans.

3.2.4. Where patients have not responded adequately to corticosteroid therapy, there is insufficient evidence to offer the addition of an anti-CCR4 monoclonal antibody

Conclusion/expert opinion: Although further clinical studies, both in Japan and elsewhere, are required to confirm the safety, efficacy, and durability of this therapy, the initial findings are promising. There are, however, insufficient data at present to recommend this therapy outside clinical trials, and the treatment is not widely available.

3.2.5. There is insufficient evidence to recommend the offer of alternative therapies (See table 2 in Full Document online, links.lww.com/CPJ/A170) as first-line therapy outside of a clinical study.

Conclusion/expert opinion: A wide range of additional therapies has been reported, which are not in current practice. These include anti-CD25 monoclonal antibody, erythromycin, heparin, immunoglobulin, *Lactobacillus casei strain Shirota*, the heparinoid, pentosan polysulfate sodium, pentoxifylline, prosultiamine, plasmapheresis, sodium valproate, and intermittent high-dose vitamin C.

Three studies have explored the potential of the anabolic steroid danazol, and further study is merited.

4. Treatment of rapidly progressing HAM/TSP

4.1. Induction therapy with pulsed methylprednisolone (1 g daily for 3–5 days) should be offered.

4.2. Alternatively, the induction treatment may include high-dose prednisolone (0.5 mg/kg daily per oral) for up to 14 days.

4.3. After the induction therapy with high-dose steroids, maintenance therapy as per Section 3 should be offered.

Rapidly progressing HAM/TSP may result in such severe bilateral lower limb paraparesis, with or without spasticity, that the patient will become totally wheelchair dependent within a few months. In such circumstances, the panel recommends early initiation of HAM/TSP disease-modifying therapy with high-dose (1 g) pulsed IV methylprednisolone for up to 5 days. Where this is not readily available, high-dose oral prednisolone can be substituted. Where no response or limited response is seen after IV pulsed methylprednisolone, further treatment with high-dose oral prednisolone for 2 weeks can be added followed by a gradual, clinically responsive reduction in dose. Panel members have observed that some patients are quite steroid sensitive and that exacerbations occur as the dose is reduced, even at doses as high as 15 mg prednisolone daily. The panel recommends that all patients with rapid progression continue with maintenance therapy and that steroids are not stopped abruptly. This can be low dose (5–10 mg daily) of oral prednisolone or steroid-sparing agents as described in Section 3. In the ciclosporin study of early or progressing disease, treatment was given for 48 weeks and then discontinued, following which some patients quickly deteriorated, whereas others maintained the clinical improvement for the 24 weeks' scheduled follow-up. In unpublished long-term follow-up, all patients eventually recommenced a disease-modifying agent due to further progression.

5. Treatment of very slowly or nonprogressing HAM/TSP

There is insufficient evidence to recommend that disease-modifying drug therapy be offered to patients with very slow or nonprogressing HAM/TSP, who have no biological evidence of disease activity.

The panel considered that there was insufficient evidence to warrant the offer of treatment with steroids or steroid-sparing agents at this time and that a watchful waiting approach, with symptomatic management and physical therapies, was sufficient. The prognostic use of biomarkers of disease activity, especially CSF cytokines, has recently been published and may become an additional decision-making tool.^5^
